# Performance of Self-Collected Saliva Testing Compared with Nasopharyngeal Swab Testing for the Detection of SARS-CoV-2

**DOI:** 10.3390/v13050895

**Published:** 2021-05-12

**Authors:** Florence Carrouel, Martine Valette, Hervé Perrier, Maude Bouscambert-Duchamp, Claude Dussart, Paul Tramini, Denis Bourgeois

**Affiliations:** 1 Laboratory “Health Systemic Process”, EA4129, University Lyon 1, 69008 Lyon, France; claude.dussart@univ-lyon1.fr (C.D.); denis.bourgeois@univ-lyon1.fr (D.B.); 2 Virology Laboratory, Institute of Infectious Agents, Croix-Rousse Hospital, Hospices Civils of Lyon, 69004 Lyon, France; martine.valette@chu-lyon.fr (M.V.); maude.bouscambert-duchamp@chu-lyon.fr (M.B.-D.); 3 Clinical Research Unit, Protestant Infirmary, 69300 Caluire-et-Cuire, France; herve.perrier@infirmerie-protestante.com; 4 Department of Public Health, Faculty of Dental Medicine, University of Montpellier, 34090 Montpellier, France; paul.tramini@orange.fr

**Keywords:** saliva, viral load, COVID-19, nasopharyngeal, SARS-CoV-2

## Abstract

The aim of this study was to determine whether self-collected pure saliva (SCPS) is comparable to nasopharyngeal (NP) swabs in the quantitative detection of SARS-CoV-2 by RT-PCR in asymptomatic, mild patients with confirmed COVID-19. Thirty-one patients aged from 18 to 85 years were included between 9 June and 11 December 2020. A SCPS sample and a NP sample were taken for each patient. Quantitative PCR was performed to detect SARS-CoV-2 viral load. Results of SCPS vs. NP samples testing were compared. Statistical analyses were performed. Viral load was significantly correlated (*r* = 0.72). The concordance probability was estimated at 73.3%. In symptomatic adults, SCPS performance was similar to that of NP swabs (Percent Agreement = 74.1%; *p* = 0.11). Thus, the salivary test based on pure oral saliva samples easily obtained by noninvasive techniques has a fair agreement with the nasopharyngeal one in asymptomatic, mild patients with a confirmed diagnosis of COVID-19.

## 1. Introduction

SARS-CoV-2 is found in nasopharyngeal (NP) secretions, and its viral load is consistently high in the saliva mainly in the early stage of the disease [1]. Saliva is considered a readily available diagnostic source for SARS-CoV-2 screening; however, biologically, the diagnostic sensitivity appears to be lower [2,3]. The sampling techniques studied are mainly based on deep-throat saliva [4], oropharyngeal swabs, and saliva from sputum after stimulation. These saliva samples are considered to be “enhanced” [5].

Certain regions of the world, such as Hong Kong, have already adopted a saliva testing outpatient program in their mass-screening protocols [6]. In February 2021, the saliva test was authorized to screen populations in France on a massive scale. The test is based on self-collected, pure oral saliva followed by PCR analysis. However, little is known about differences in the performance of self-collected pure saliva (SCPS) versus NP sampling tests, especially in asymptomatic, mild, outpatient SARS-CoV-2.

The objective of this study was to determine whether SCPS is comparable to NP swabs in the quantitative detection of SARS-CoV-2 by RT-PCR in patients with confirmed COVID-19.

## 2. Materials and Methods

### 2.1. Subjects and Specimens

All 18 to 85-year-old patients with confirmed COVID-19 infection, asymptomatic and mild clinical symptoms, and clinical signs for less than 8 days were eligible for participation in this prospective study. Asymptomatic patients are defined as individuals without clinical signs whereas mild corresponds to outpatients and patients with clinical symptoms without pneumonia manifestation through image results [7]. Between 9 June and 11 December 2020, patients with qualitative laboratory-confirmed COVID-19 were included in this study [8]. The selected population was ambulatory adults who had voluntarily presented for a screening qualitative PCR test to the Hospital Center, Le Puy en Velay, France.

Exclusion criteria were pregnancy, breastfeeding, inability to comply with protocol, lack of written agreement, patients using mouthwash on a regular basis (more than once a week), patients at risk of infectious endocarditis, patients unable to answer questions, and uncooperative patients.

After inclusion, the patients were re-tested simultaneously with both nasopharyngeal swabs and salivary collection at the same time point (9 a.m.), performed by an accredited health care professional. One NP sample and one salivary sample were obtained. The NP swab was taken in the opposite nostril from the one swabbed during the initial screening test. The saliva sample was collected using the Saliva Collection System kit (Greiner Bio-one, Kremsmünster, Austria). The patient rinsed the oral cavity with the saliva extraction solution for 2 min. The pure, non-stimulated saliva was collected by simple spitting, without effort of coughing or throat clearing, into a saliva-collection beaker. The saliva sample (between 1 and 3.5 mL) was then transferred to a tube and stored at 4 °C until analysis. Demographic data and clinical characteristics were collected from the participants and recorded in an electronic medical record (e-CRF).

The ethics committee Comité de Protection des Personnes Sud Mediterranee III (University Hospital Center of Nîmes, Nîmes, France) approved the study protocol. Written informed consent was obtained from all patients. The study was done in accordance with the principles of the Declaration of Helsinki and the International Conference on Harmonization–Good Clinical Practice guidelines.

### 2.2. Quantification of Viral Load

RNA extraction was performed by the laboratories of the National Reference Center for Respiratory Viruses, France, using the NucliSens easyMAG instrument (bioMérieux, Marcy-l’Etoile, France) following the manufacturer’s instructions. RdRp-IP2 and RdRp-IP4 quantitative RT-PCR was used to detect SARS-CoV-2. Primer and probe sequences (Eurofins, Genomics, Germany), described in the Table 1, correspond to the RdRp-IP2 and the RdRp-IP4 assays designed at The Institut Pasteur to target a section of the RdRp gene (nt 12621-12727 and 14010-14116 positions) based on the sequences of SARS-CoV-2 (NC_004718) made available on the Global Initiative on Sharing All Influenza Data database on 11 January 2020.

Real-time RT-PCR was performed with the Invitrogen SuperscriptTM III Platinum One-Step qRT-PCR system (Invitrogen, Illkirch, France). The mix contained 5 μL of RNA, 1 μL of RdRp-IP2 forward primer (0.4 μM), 1 μL of RdRp-IP2 reverse primer (0.4 μM), 1 μL of RdRp-IP4 forward primer (0.4 μM), 1 μL of RdRp-IP4 reverse primer (0.4 μM), 12.5 μL of 2X reaction buffer, 1 μL of Superscript III RT/Platinum Taq Mix, and 0.4 μL of a 50 mM magnesium sulfate solution. The assays were performed on a Quant Studio 5 (Thermofisher, Dardilly, France) with the following program: 55 °C for 10 min (reverse transcription), followed by 95 °C for 2 min, and then 45 cycles of 95 °C for 3 s and 58 °C for 30 s. Each run included three negatives samples bracketing unknown samples during RNA extraction, two positive controls, and one negative amplification control. When a sample was positive for RdRp-IP4, the quantification of the number of RNA copies was performed according to a scale ranging from 10^2^ to 10^6^ copies per μL. The quality of the nasopharyngeal swabs was assessed using the CELL Control r-gene kit (bioMérieux). The cellular load was measured using this kit. This kit is provided with a quantified plasmid for human cellular quantification (alive or dead cells) targeting the HRT-1 gene. From a single test sample, the viral load [9] and the quantity of cells was determined, and the final result can be expressed as log_10_ copies per 10,000 cells. The viral load in saliva was calculated as the number of RNA copies per mL of saliva.

### 2.3. Statistical Analysis

The samples were divided respectively according to the SARS-CoV-2 viral load and the nature of the symptoms. The difference in the proportion of positive NP swabs and SCPS for SARS-CoV-2 RNA was assessed using Fisher’s exact test. To determine whether SCPS swabs were comparable to NP samples in detecting SARS-CoV-2 at lower viral load values, NP swabs were categorized into 2 groups based on swab median values. The correlation between NP and SCPS viral load values was determined by the Spearman rank correlation test and represented graphically with linear regression. Copy numbers were correlated by pairs between different groups. Statistical analyses were conducted using R (v3.6.0, The R Foundation for Statistical Computing Platform).

## 3. Results

### 3.1. Characteristics of COVID-19 Patients

Thirty-one patients were included. The mean age was 43.0 ± 15.5 years, ranging from 22 to 75 years, and 48.4% of patients were male. A total of 96.8% were outpatients, and 74.2% of participants had no medical antecedents. In total, 9.7% of patients had no clinical signs, and 90.3% presented mild symptoms with 4.10 ± 1.41 symptoms of the reported forms of COVID-19. Presenting complaints included dyspnea (7%), fever (42.9%), febrile symptoms (16.7%), asthenia (60%), cough (71%), myalgias (57%), gastrointestinal symptoms (14%), headache (50%), anosmia (50%), and ageusia (39%). NP and SCPS specimens were collected at a median time of 4 days (95% CI; 3–5 days) after the initial qualitative screening for NP PCR. Samples were collected at a median of 6 days (95% CI; 5–7 days) after symptom onset.

### 3.2. Comparison of Salivary and Nasopharyngeal SARS-CoV-2 Viral Load

**Figure 1:**
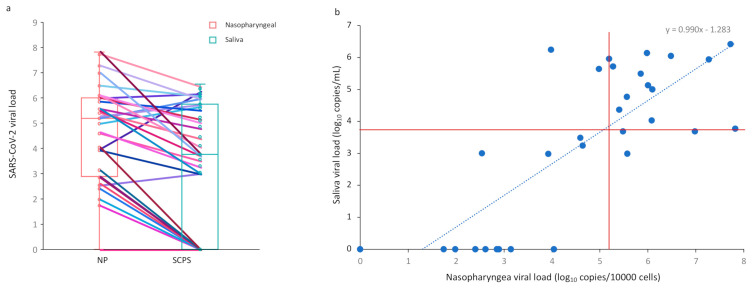
Analysis of SARS-CoV-2 viral load in nasopharyngeal (log_10_ copies/10,000 cells) and saliva (log_10_ copies/mL) samples. (**a**) Repartition of SARS-CoV-2 values for nasopharyngeal and saliva specimens. Pairs for each subjects are connected by lines; (**b**) SARS-CoV-2 values by testing concordance. The saliva SARS-CoV-2 value was set to 30 for samples in which only the nasopharyngeal target was detected. Red horizontal and vertical lines indicate the median. The median for each group was 5.27 log_10_ copies/10,000 cells and 3.69 log_10_ copies/mL for nasopharyngeal and saliva samples, respectively. NP, nasopharyngeal; SCPS, self-collected pure saliva.

The NP sample was reported to be in the range of 3.53–5.99 log_10_ SARS-CoV-2 copies/10,000 cells, with a median of 5.27 log_10_ copies/10,000 cells. The saliva sample was reported to be in the range of 0.0–5.56 log_10_ SARS-CoV-2 copies/mL, with a median of 3.69 log_10_ copies/mL (Figure 1a).

Among the 31 patients included, one had no detectable NP or SCPS viral load. The results of the NP and SCPS tests in pairs are presented in Figure 1b. The performance of SCPS versus NP samples was compared against the total number of positives regardless of specimen type. The overall concordance for saliva was 73.3% (22/30) with saliva yielding detection of 8 fewer cases than NP (*p* = 0.005). A total of 72.7% (16/30) of pairs were positively matched (nasal viral load > salivary viral load), and 18.2% (4/20) of pairs were negatively concordant. The percentage of agreement (PA) was 74.1% (*p* = 0.11) for symptomatic adults and 66.7% (*p* = 1) for asymptomatic cases.

A significant correlation between NP and SCPS samples was observed between the two tests (*r* = 0.72, *p* = 0.0001) (Figure 1b). For patients with an NP viral load greater than 5.2 log_10_/10,000 cells (median value), the corresponding saliva samples were positive (15/15 [PA = 100%]). On the other hand, for the patients with NP viral loads less than 5.2 log_10_ copies/10,000 cells, only 7 of the 17 SCPS samples were positive (PA = 41.2%, *p* = 0.007) (Figure 1b). Taking into account the first quartile (NP viral load less than 3.0 log_10_ copies/10,000 cells), only one SCPS sample was positive among the seven positive NP samples (PA = 14.3%, *p* = 0.005).

### 3.3. Correlation between SARS-CoV-2 Viral Load and COVID-19 Symptoms

**Figure 2:**
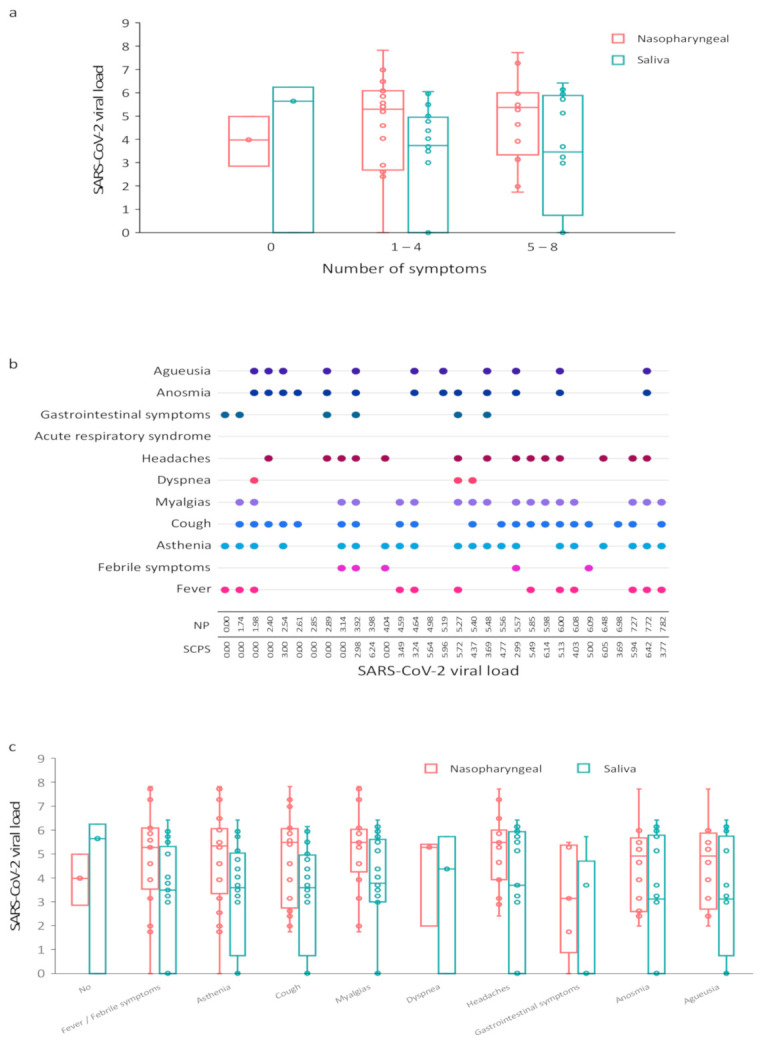
Correlation between SARS-CoV-2 viral load and COVID-19 symptoms. (**a**) SARS-CoV-2 load according to the number of clinical symptoms; (**b**) Symptoms according to the SARS-CoV-2 load for each subject; (**c**) SARS-CoV-2 load according to the type of clinical symptoms. NP, nasopharyngeal; SCPS, self-collected pure saliva.

Among the 31 pairs of samples available, viral loads were not significantly correlated for days one through five (*r* = 0.52, *p* = 0.18) after symptom onset and then significantly correlated for days six through seven (*r* = 0.73, *p* < 0.0001) and day seven (*r* = 0.83, *p* = 0.002). Among these available NP and saliva samples, viral loads were significantly correlated between the two sample types for the number of symptoms one through four (*r* = 0.60, *p* = 0.007) and five through eight (*r* = 0.89, *p* < 0.0001) (Figure 2a). Symptoms according to the SARS-CoV-2 load for each subject are described in Figure 2b,c. Three patients had no symptoms. The cluster of musculoskeletal symptoms encompassed headache, myalgia, fatigue, and joint pain. These were frequent symptoms because they affected 26 of the 28 symptomatic patients. Headache was observed in 15 of 28 symptomatic COVID-19 outpatients. Fever, febrile symptoms, cough, and shortness of breath symptoms occurred in the majority of cases (21 of 28). Fever was observed with a median maximum of 38.2 °C. No patient had a temperature higher than 39 °C. The most cough occurred in 20 of 28 symptomatic patients. Otolaryngeal symptoms, including anosmia and agueusia, were important signs of the disease (24 of 28). Six of the 28 symptomatic patients had gastrointestinal symptoms.

**Figure 3:**
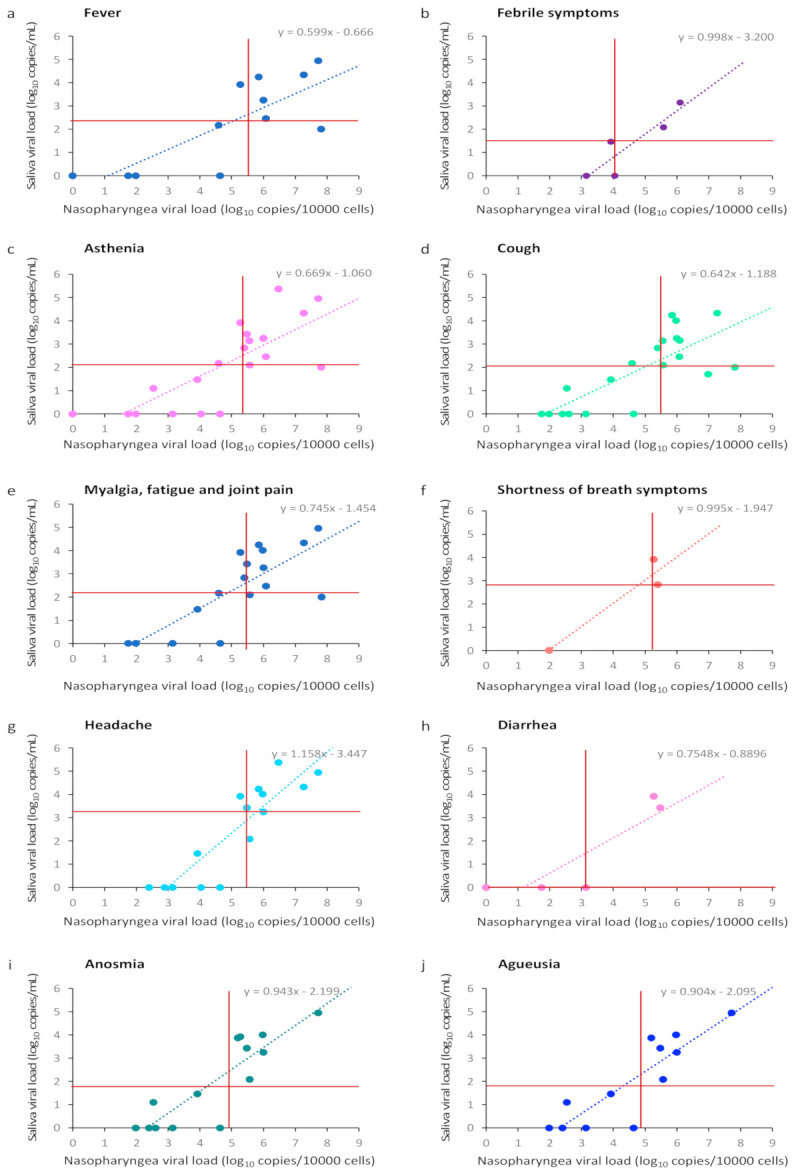
Analysis of SARS-CoV-2 viral load in nasopharyngeal (log_10_ copies/10,000 cells) and saliva (log_10_ copies/mL) samples according to clinical symptoms. (**a**) SARS-CoV-2 values by testing concordance for fever; (**b**) SARS-CoV-2 values by testing concordance for febrile symptoms; (**c**) SARS-CoV-2 values by testing concordance for asthenia; (**d**) SARS-CoV-2 values by testing concordance for cough; (**e**) SARS-CoV-2 values by testing concordance for myalgia, fatigue, and joint pain; (**f**) SARS-CoV-2 values by testing concordance for headache; (**g**) SARS-CoV-2 values by testing concordance for diarrhea; (**h**) SARS-CoV-2 values by testing concordance for anosmia; (**i**) SARS-CoV-2 values by testing concordance for anosmia; (**j**) SARS-CoV-2 values by testing concordance for agueusia. Red horizontal and vertical lines indicate the median.

Among the 28 pairs of samples available, viral loads were significantly correlated for fever (*r* = 0.78, *p* < 0.001), febrile symptoms (*r* = 0.90, *p* < 0.001), asthenia (*r* = 0.78, *p* < 0.001), cough (*r* = 0.78, *p* < 0.001), myalgia, fatigue and joint pain (*r* = 0.78, *p* < 0.001), shortness of breath symptoms (*r* = 0.95, *p* = 0.03), headache (*r* = 0.89, *p* < 0.001), diarrhea (*r* = 0.87, *p* = 0.03), anosnia (*r* = 0.87), and agueusia (*r* = 0.87, *p* < 0.001). (Figure 3).

## 4. Discussion

Our study of coupled sampling of NP and SCPS from a population of confirmed COVID-19 outpatients with asymptomatic to mild symptoms indicated that samples are reliable for outpatient diagnosis. Furthermore, the time elapsed in the first week after the onset of symptoms did not affect the consistency of the tests. However, SCPS samples were less effective at detecting SARS-CoV-2 in patients with a low viral load in the upper respiratory tract. Concordance between SCPS and NP tests increased with the amount of nasopharyngeal viral load and, by extension, perhaps with greater infectivity [10].

Although the current standard involves testing NP swab samples by PCR for SARS-CoV-2, this sample collection is a relatively invasive method [11]. It is not suitable for all situations, especially when the test must be repeated or if NP collection is difficult or impossible. The objective of salivary tests is to facilitate sampling, reduce the risk of contamination of health care personnel, and provide a less unpleasant test for patients, particularly for children or for large-scale targeted screening: schools, universities, health care personnel [2,12,13]. Similarly, they reduce the need for personal protective equipment and swabs. Finally, the analysis of RNA concentration variations during clinical course has shown less important fluctuations in saliva than in NP samples [14].

If studies have shown that SARS-CoV-2 can be detected in the saliva of asymptomatic and outpatients [11,13], a few studies compared viral load concordance rate between nasopharyngeal and pure, oral saliva collection in ambulatory adults with no-or mild symptoms associated to a real-time reverse transcription-quantitative PCR analysis [5]. Huber et al. observed, for instance, a good positive percent agreement of enhanced saliva and NPS in symptomatic (mild/strong) adults (PPA = 92.9%). However, individuals were asked to clear their throat one to three times thoroughly and collect saliva one or two times into the same tube. Similarly, Pasomsub et al. observed in 33 COVID-19 adults a concordance of 97.5% between NP and salivary testing using a salivary throat collection [11]. Migueres et al. reported 44 infected hospitalized and ambulatory individuals comprised 34 (77.3%) with both samples positive [10]. However, information on symptomatic or asymptomatic status were not specified.

The well-conducted study by Iwasaki et al. examined the efficacy of PCR detection of SARS-CoV-2 between paired nasopharyngeal and saliva samples in 76 patients with mild to moderate disease with no patient requiring ventilator, including ten COVID-19 patients [15]. SARS-CoV-2 was detected in eight of ten patients in both NP and saliva samples, and in either sample only in two of ten patients. The overall concordance rate of the virus detection between the two samples reached as high as 97.4%. In our mild symptomatic ambulatory patient, percent agreement (74.1%) were less consistent to these data. The differences observed between the two studies concerning the median age of the samples, the median number of days associated with nasal and salivary collection after symptom onset, and the number of patients should be taken into consideration when comparing these results [16,17]. Similarly, data regarding the nature of symptoms as well as nasal and salivary viral load would merit comparative investigation.

Patients with no symptoms at the time of collection had lower NP and SCPS viral load values than those with symptoms. Although the saliva load is lower, the tests can be performed with a lower concordance rate [18]. In individuals who have a negative SCPS test for SARS-CoV-2, it may be necessary to consider the signs and symptoms of COVID-19 and perform a complementary NP test [19].

Two meta-analyses focused on the use of saliva for the detection of SARS-CoV-2 RNA and found differences among the studies [20,21]. Variations in the diagnostic accuracy of saliva tests are due to the population studied, the timing of tests and specimen collection, the nature of the saliva sample collection (i.e., stimulation, oropharynx, sputum, throat washing), patient symptoms, and the timing of testing and nasopharyngeal sampling [21]. Moreover, discordance in the exact type of saliva specimen used in these research papers exists [6].

Our study has some limitations. First, the sample size of this research is relatively small, which can lead to some correlations not being detected. Second, only COVID-19-positive patients were included. Therefore, there is no possibility with the data collected from this study to evaluate the presence of any false positives, the sensitivity, the specificity, and the accuracy of the test.

## 5. Conclusions

Our conclusion is that the salivary test based on pure, oral saliva samples easily obtained by noninvasive techniques has a fair agreement with the nasopharyngeal one in patients with a confirmed diagnosis of COVID-19.

## Figures and Tables

**Table 1:** Real-time RT-PCR primers and probes for the detection of SARS-CoV-2.

Name	Sequences (5′-3′)	PCR Product
***RdRp gen/nCoV_IP2***		108 pb
nCoV_IP2-12669Fw ^1^	ATGAGCTTAGTCCTGTTG	
nCoV_IP2-12759Rv ^1^	CTCCCTTTGTTGTGTTGT	
nCoV_IP2-12669bProbe (+) ^1^	[5′]HEX-AGATGTCTTGTGCTGCCGGTA-[3′]BHQ-1	
***RdRp gene/nCoV_IP4***		107 pb
nCoV_IP4-14059Fw ^1^	GGTAACTGGTATGATTTCG	
nCoV_IP4-14146Rv ^1^	CTGGTCAAGGTTAATATAGG	
nCoV_IP4-14084Probe (+) ^1^	[5′]Fam-TCATACAAACCACGCCAGG-[3′]BHQ-1	

^1^ National Reference Center for Respiratory Viruses, Institut Pasteur, Paris, France.

## Data Availability

The data presented in this study are available on request from the corresponding author.
